# Early brain computed tomography findings are associated with outcome in patients treated with therapeutic hypothermia after out-of-hospital cardiac arrest

**DOI:** 10.1186/1757-7241-21-57

**Published:** 2013-07-19

**Authors:** Soo Hyun Kim, Seung Pill Choi, Kyu Nam Park, Chun Song Youn, Sang Hoon Oh, Se Min Choi

**Affiliations:** 1 Department of Emergency Medicine, College of Medicine, The Catholic University of Korea, Seoul, South Korea; 2 Department of Emergency Medicine, Uijeongbu St. Mary’s Hospital, Geumo-dong, Uijeongbu-si, Gyeonggi-do, 480-717, Republic of Korea

**Keywords:** Cardiac arrest, Brain computed tomography, Therapeutic hypothermia, Prognosis

## Abstract

**Background:**

This study evaluated the association between the results of immediate brain computed tomography (CT) scans and outcome in patients who were treated with therapeutic hypothermia after cardiac arrest. The evaluation was based on the changes in the ratio of gray matter to white matter.

**Methods:**

A total of 167 patients who were successfully resuscitated after cardiac arrest from March 2009 to December 2011 were included in this study. We selected 51 patients who received a brain CT scan within 1 hour after the return of spontaneous circulation (ROSC) and who had been treated with therapeutic hypothermia. Circular regions of measurement (10 mm^2^) were placed over regions of interest (ROIs), and the average attenuations in gray matter (GM) and white matter (WM) were recorded in the basal ganglia, at the level of the centrum semiovale and in the high convexity area. Three GM-to-WM ratios (GWRs) were calculated: one for the basal ganglia, one for the cerebrum and the average of the two. The neurological outcomes were assessed using the Cerebral Performance Category (CPC) scale at the time of hospital discharge, and a good neurological outcome was defined as a CPC score of 1 or 2.

**Results:**

The average GWR was the strongest predictor of poor neurological outcome as determined using receiver operating characteristic curves (basal ganglia area under the curve (AUC) = 0.716; cerebrum AUC = 0.685; average AUC = 0.747). An average GWR < 1.14 predicted a poor neurological outcome with a sensitivity of 13.3% (95% confidence interval (CI) 3.8-30.7), a specificity of 100% (95% CI 83.9-100), a positive predictive value of 100% (95% CI 2.5-100), and a negative predictive value of 44.7% (CI 28.9-58.9).

**Conclusions:**

Our study demonstrated that low GWRs in the immediate brain CT scans of patients treated with therapeutic hypothermia after ROSC were associated with poor neurological outcomes. Immediate brain CT scans could help predict outcome after cardiac arrest.

## Background

Cardiac arrest is a significant public health problem. Over 300,000 cases of out-of-hospital cardiac arrest occur in North America annually, and the patient fatality rate approaches 95% [1]. The treatment of out-of-hospital cardiac arrest (OHCA) survivors has changed dramatically over the past decade. Aggressive therapeutic interventions, such as therapeutic hypothermia and thrombolysis, implemented immediately after resuscitation have been attempted to improve the outcomes for OHCA [2-4].

Brain injury significantly contributes to mortality in patients after cardiac arrest. The majority of patients who are resuscitated after cardiac arrest are comatose due to ischemic brain injury. The clinicians who treat comatose patients after cardiac arrest have struggled with difficult decisions about the continued provision of intensive care. Therefore, accurate methods for predicting patient outcomes at the earliest time are required. Several diagnostic modalities may predict the outcome of OHCA survivors, including the use of blood markers and electrophysiological studies [5,6].

A previous study reported that in contrast to Europe, SAH is more frequently found in Asia, up to 16.2% of the OHCA survivors [7]. After the resuscitation of comatose patients, an immediate computed tomography (CT) scan is one of the most frequently used ancillary imaging modalities to identify brain lesions such as stroke, intracranial hemorrhage and to decide whether therapeutic hypothermia is indicated. CT scans are easily obtained in comatose cardiac arrest patients, and anesthetic agents or other treatments do not influence the results. Therefore, CT scans are frequently ordered to evaluate comatose patients after cardiac arrest.

Cerebral edema is a marker of severe brain injury associated cardiac arrest. This type of edema appears as a loss of differentiation between gray matter (GM) and white matter (WM) on brain CT images. Several previous studies have found that the level of differentiation between the GM and the WM is significantly lower in post-cardiac arrest patients with poor outcomes than in patients with good outcomes [8,9]. However, following implementation of therapeutic hypothermia, as with other diagnostic modalities, the ability of brain CT to predict outcome in patients after cardiac arrest needs to be reassessed.

We evaluated the association between the CT signs and the outcomes of patients treated with therapeutic hypothermia after cardiac arrest. This evaluation was based on the decrease in the GM to WM ratio (GWR) obtained from a brain CT scan performed within 1 hour after return of spontaneous circulation (ROSC).

## Materials and methods

### Patients

This retrospective observational study was conducted in a tertiary urban educational hospital, the Seoul St. Mary’s Hospital, from March 2009 to December 2011. Our institutional review board approved this study. Inclusion criteria were an age >18 years, out-of-hospital cardiac arrest unrelated to trauma and the return of spontaneous circulation. We identified subjects who had obtained a brain CT scan immediately (within 1 hour) after ROSC, including transfer patients from outside hospitals and patients who were treated with therapeutic hypothermia. In accordance with our institutional protocol for post cardiac arrest care, comatose patients who were successfully resuscitated received brain CT scans immediately to determine the cause of arrest. Therapeutic hypothermia (TH) was initiated as soon as possible using bags of ice and the infusion of 4°C normal saline simultaneously with other procedures, including CT scans. Patients whose CT scans indicated parenchymal abnormalities, patients who did not complete the therapeutic hypothermia process due to hemodynamic instability or patients for whom CT images were not available for analysis were excluded from the analysis.

### Therapeutic hypothermia

All unconscious patients who were resuscitated from nontraumatic OHCA and brought to the emergency department were eligible for the TH. Patients were excluded from the TH according to the following criteria: evidence of significant active bleeding, any intracranial hemorrhage, hemodynamic instability unresponsive to volume resuscitation and vasopressor treatment, severe dysrhythmia unresponsive to antiarrhythmic therapy, known terminal illness, poor prearrest neurologic status, and those with a “do not attempt resuscitation” preference. Ultimately, deciding whether a TH was initiated was up to the discretion of the treating physicians.

To induce TH, patients were covered with ice bags over the neck, axillae, torso, and groins. In addition, TH was induced by rapid intravenous infusion of 1–2 L of 4°C saline, evaporative cooling by moist fanning, fully exposing patient, an endovascular cooling device (Thermogard XP^®^ Thermal Management System; Zoll, Sunnyvale, CA) or some combination of these techniques. An arterial line was inserted before TH induction. Patients were given sedation (midazolam), analgesia (fentanyl), and a paralyzing agent (rocuronium). Neuromuscular blockade was discontinued when the goal temperature was reached, but if shivering was not controlled with the sedative and analgesic agents, the continuous infusion of a neuromuscular blocker was restarted. These agents were stopped during rewarming as soon as the central temperature reached 35°C. The goal was to achieve a temperature of 32–34°C as soon as possible and to maintain it for 24 hours. The endovascular cooling device was adjusted manually to keep temperatures close to a goal of 33°C. Rewarming was then started at a rate of 0.25°C/h with absolute avoidance of any active measure, first to a target of 36°C and then ensuring that 37.5°C was not exceeded and using an endovascular cooling device, until 72 hours had elapsed from the return of spontaneous circulation (ROSC).

### Data collection

Medical records were collected for the patients who were included this study. The following demographic and clinical data were collected for each patient: age, sex, underlying disease (e.g., hypertension, diabetes, coronary artery disease, renal disease, pulmonary disease, stroke), cause of arrest, witnessed collapse, bystander cardiopulmonary resuscitation (CPR), initially presenting rhythm after arrest, resuscitation time, time from ROSC to CT obtained, Glasgow Coma Scale (GCS) score, neurological examination on admission, discharge status and Cerebral Performance Category (CPC) scale at the time of hospital discharge. The neurological outcomes of the patients were assessed using the CPC scale at the time of hospital discharge, and a good neurological outcome was defined as a CPC score of 1 or 2.

### CT measurements

**Figure 1:**
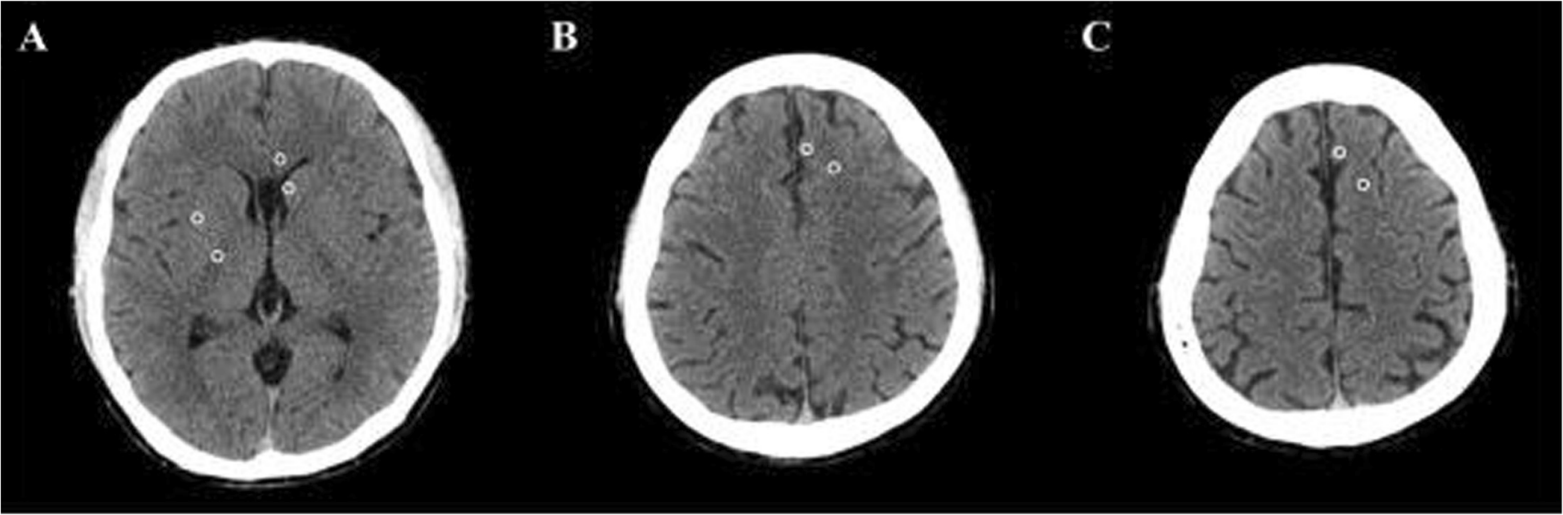
**Brain computed tomography images showing measurements in Hounsfield units at the basal ganglia level (left), the centrum semiovale level (middle), and the high convexity level (right).** The circular regions of interest (10 mm2) are positioned in the caudate nucleus, putamen, posterior limb of the internal capsule, and genu of the corpus callosum at the level of the basal ganglia and are positioned in the medial cortex and medial white matter at the centrum semiovale and high convexity levels.

Only non-contrast brain CT scans were performed. All of the recorded CT scans that were taken at the time of admission to the emergency department were included in this study. A 64-channel scanner (LightSpeed VCT; GE HealthCare, Milwaukee, Wisconsin, USA) was used for all of the CT studies with a 5-mm slice width. Two investigators, who were blinded to the clinical data, independently accessed the CT scan slices for each patient’s brain at the level of the basal ganglia, defined as the image in which the caudate nucleus, internal capsule, third ventricle, and sylvian fissures were visualized, and at two levels of the superior cortex, as described previously: (1) the centrum semiovale level, defined as the image 5 mm above the lateral ventricular system, and (2) the high convexity level, defined as the next image 5 mm above the centrum semiovale level [8,9]. The measurement cursor was configured as a 10 mm^2^ circular surface. Circular regions of measurement were placed over the regions of interest (ROI) in the right and left sides, and the average attenuation in Hounsfield Units (HU) was recorded. The values at the basal ganglia level were recorded in the caudate nucleus (CN), putamen (PU), corpus callosum (CC), and the posterior limb of the internal capsule (PIC). The values at the level of the centrum semiovale were recorded in the medial cortex (MC1) and the medial white matter (MW1). The values at the high convexity area were recorded using the same method (MC2 and MW2, respectively). We calculated three GWRs: the basal ganglia, cerebrum and average GWRs [10]. The GWRs were calculated according to the following formulas (Figure 1):

GWR Basal ganglia = (CN + PU)/(CC + PIC)

GWR Cerebrum = (MC1 + MC2)/(MW1 + MW2)

GWR Average = (GWR Basal ganglia + GWR Cerebrum)/2

### Statistical analyses

Categorical data are expressed as frequencies and percentages. We tested the distributions of the continuous variables (raw HU measurements and GWR) for normality using visual inspection and the Shapiro-Wilk test. Normally distributed data are expressed as the means and standard deviations using Student’s-*t* test. Non-normally distributed data were assessed using the Mann–Whitney *U* test. Chi-squared tests were used to identify differences in patient demographic data between the two groups. We examined the receiver operating characteristic (ROC) curves to determine the optimal (maximum specificity) threshold value of the GWR for the prediction of poor neurological outcomes. We expressed the performance of each measure for the prediction of poor neurological outcomes as the area under the curve (AUC) for the ROC curves. We determined the sensitivity, specificity, positive predictive value and negative predictive value at the optimal cutoff, which was selected from each ROC curve. All statistical analyses were performed using SPSS software, version 17.0 (SPSS, Chicago, IL, USA). Values of *P* < 0.05 were considered statistically significant for all comparisons.

## Results

### Patient demographics

**Figure 2:**
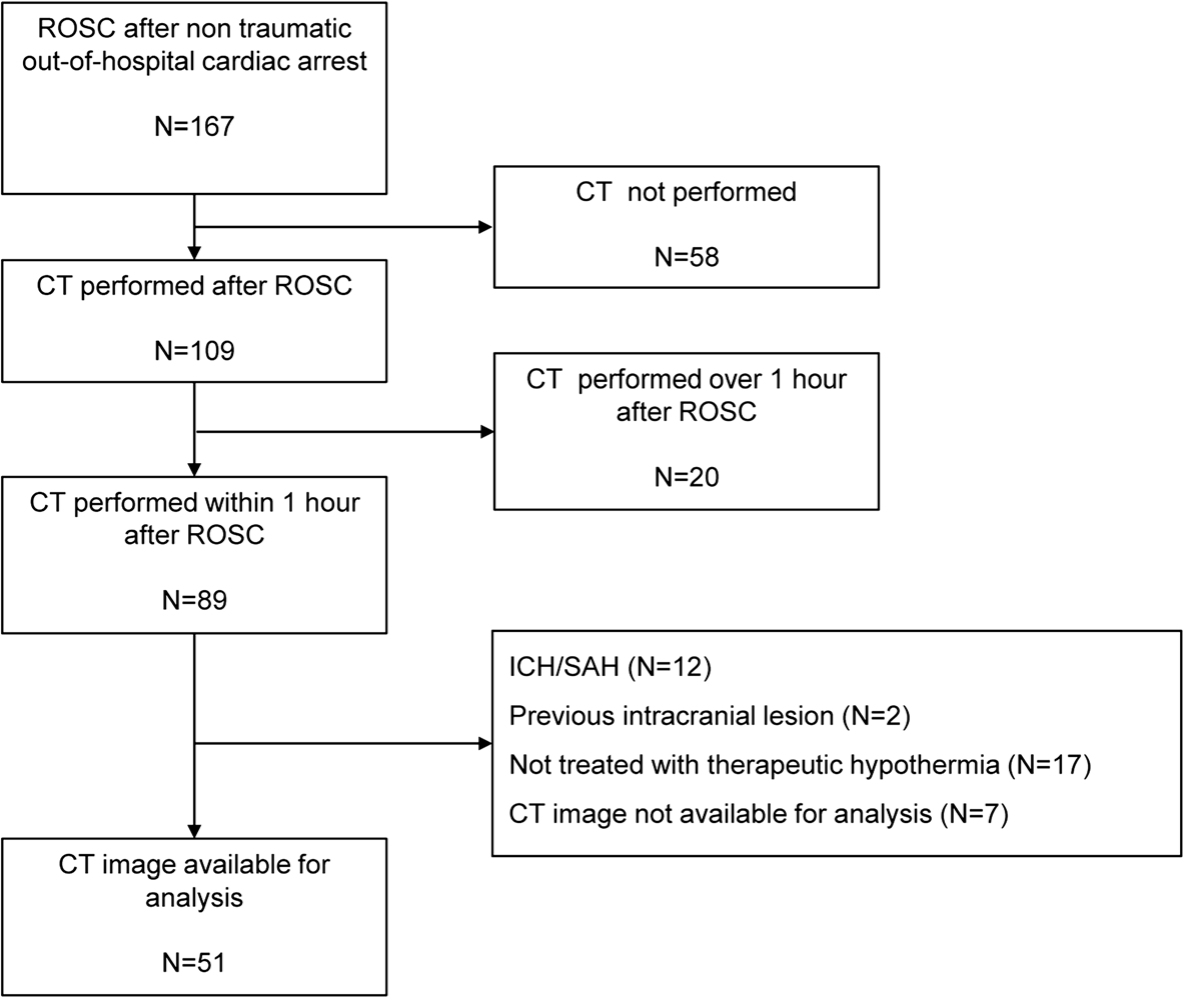
Subjects included in the study.

A total of 333 adult patients who suffered non-traumatic out-of-hospital cardiac arrest were admitted to our emergency department between January 2009 and December 2011. A total of 167 of these patients recovered spontaneous circulation after resuscitation, and post-cardiac arrest care was initiated. OHCA survivors (N = 109) underwent brain CT scans. Eighty-nine of the 109 survivors received an immediate brain CT within 1 hour. Twelve of the 89 survivors (13.5%) who received an immediate brain CT exhibited intracranial hemorrhages (especially subarachnoid hemorrhages). We excluded subjects with previous intracranial lesions (N = 2) and subjects in whom post-cardiac arrest care was withdrawn, in whom the hemodynamic status was not sustained during therapeutic hypothermia or for whom therapeutic hypothermia was not indicated (N = 17). We also excluded CT images that were not available for analysis, including CT scans that were performed at an outside hospital (N = 7). A total of 51 CT scans were analyzed (Figure 2).

The demographic data of the enrolled patients are summarized in Table 1. The mean age was 53.9 years old, and 66.7% of the patients were male. The median time to CT after ROSC was 16.0 minutes. All of the patients included in this study were comatose, with GCS scores below 6 points. In 27.5% and 21.6% of patients, respectively, pupillary light reflexes and corneal reflexes were present immediately after ROSC. Twenty-nine (56.9%) patients survived and were discharged from the hospital. Twenty-one (41.2%) of these patients achieved good neurologic outcome. (CPC1 = 18, CPC2 = 3). In most of pre arrest baseline variables, there are no significant differences between the two groups. However, there are significant differences in some variables associated with resuscitation such as witnessed, cardiac etiology and post-resuscitation neurologic exams.

**Table 1:** Baseline characteristics of the patients included in this study

	**Good outcome (n = 21)**	**Poor outcome (n** **= 30)**	***p***
Age, years, mean ± SD	55.3 ± 14.6	51.3 ± 16.8	0.365
Male, N (%)	17 (56.7)	16 (76.2)	0.200
Underlying disease, N (%)			
Hypertension	2 (9.5)	7 (23.3)	0.203
Diabetes	2 (9.5)	4 (13.3)	0.678
Stroke	0	2 (6.7)	0.227
Coronary disease	4 (19.0)	1 (3.3)	0.063
Renal disease	0	1 (3.3)	0.398
Pulmonary disease	0	4 (13.3)	0.081
Witnessed arrest, N (%)	16 (53.3)	19 (90.5)	0.005
Bystander CPR, N (%)	11 (36.7)	12 (57.1)	0.100
Shockable rhythm, N (%)	5 (16.7)	15 (71.4)	<0.001
Cardiac etiology, N (%)	15 (50.0)	20 (95.2)	0.001
Time, min, median (IQR)			
collapse to CPR start	1.0 (0.0-6.0)	7.5 (3.75-21.0)	0.009
CPR start to ROSC	20.0 (12.0-31.5)	23.5 (19.0-30.0)	0.276
collapse to ROSC	25.0 (19.0-35.5)	34.5 (25.8-48.3)	0.018
Epinephrine dose during resuscitation, median (IQR)	3.0 (2.0-4.0)	2.0 (0.0-5.0)	0.388
Time from ROSC to CT, min, median (IQR)	15.5 (6.8-25.8)	13.0 (6.5-21.5)	0.619
Neurologic examination after ROSC, N (%)			
GCS scores 3	14 (66.7)	30 (100.0)	0.009
GCS scores 4	1 (4.8)	0	
GCS scores 5	3 (14.3)	0	
GCS scores 6	3 (14.3)	0	
Present pupillary light reflex	10 (47.6)	4 (13.3)	0.007
Present corneal reflex	9 (42.9)	2 (6.7)	0.002
Present motor response	8 (38.1)	0	<0.001
Outcome			
Length of hospital stay, days, median (IQR)	16.0 (13.0-29.0)	4.0 (3.0-7.3)	<0.001
Survival discharge, N (%)	21	8	

*SD* standard deviation, *CT* computed tomography, *ROSC* return of spontaneous circulation, *OHCA* out-of-hospital cardiac arrest, *IQR* interquartile range, *CPR* cardiopulmonary resuscitation, *GCS* Glasgow coma scale.

### Measurement of attenuation in CT image

**Figure 3:**
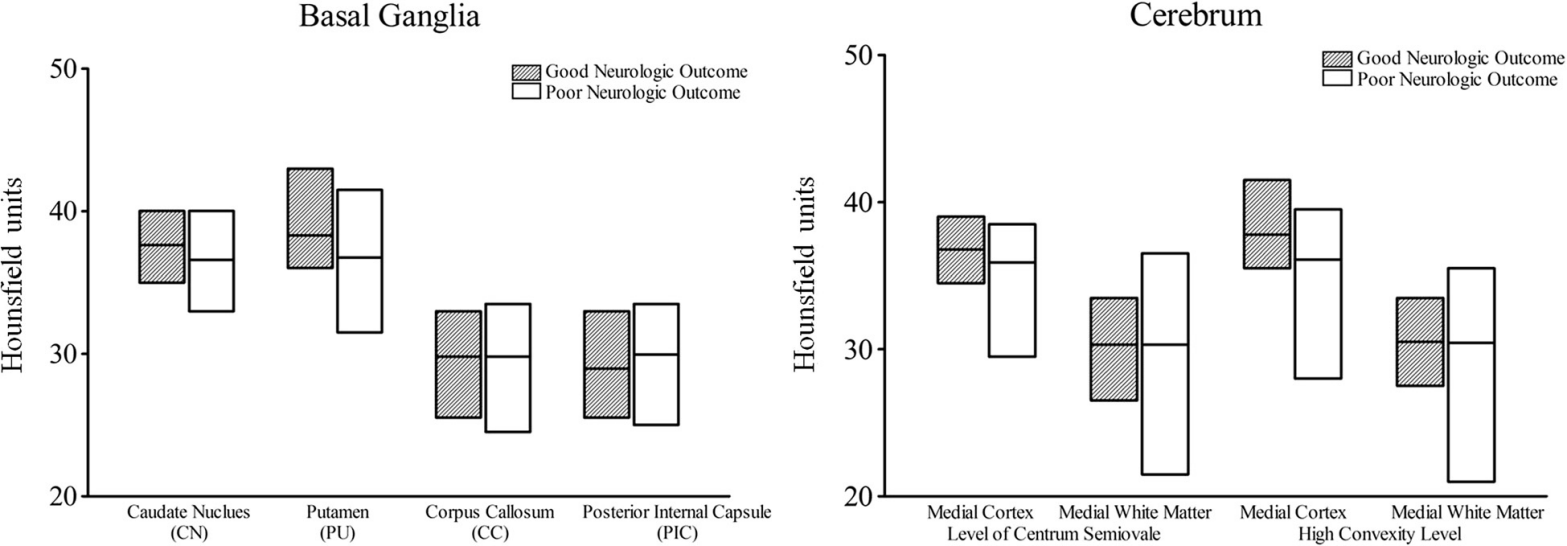
**Comparison of the attenuations in gray and white matter between groups. **The ends of the floating columns indicate the min and max values, and the middle line is the mean.

In the good outcome group, the GM attenuations were greater than in the poor outcome group. However, the WM attenuations were similar between the two groups. The GM attenuations at the basal ganglia level (CN and PU) and in the medial cortex at the high convexity level (MC2) were significantly different between the good and poor neurological outcome groups (*P* = 0.030, 0.038 and 0.043, respectively), but the GM attenuations in the medical cortex at the centrum semiovale level (MC1) were not significantly different. None of the WM attenuations (CC, PIC, MW1, and MW2) differed between the two groups (Table 2, Figure 3). The inter-rater reliability of the attenuation measurements between the two investigators exhibited a high Pearson’s correlation coefficient of 0.965 (*P* < 0.001), which indicated a strong correlation.

**Table 2:** **Comparisons of the attenuations in Hounsfield units in regions of interest and of the gray matter**-**to**-**white matter ratio between groups**

			**Good outcome (N** **=** **21)**	**Poor outcome (N** **=** **30)**	***P***
Basal ganglia	Gray matter	CN	37.6 ± 1.4	36.6 ± 1.8	0.030
		PU	38.3 ± 1.7	36.7 ± 2.5	0.038
	White matter	CC	29.8 ± 2.0	29.8 ± 2.2	0.946
		PIC	29.0 ± 2.0	29.9 ± 2.2	0.094
Centrum semiovale	Gray matter	MC1	36.8 ± 1.3	35.9 ± 2.0	0.072
	White matter	MW1	30.3 ± 1.9	30.3 ± 3.2	0.990
High convexity area	Gray matter	MC2	37.8 ± 1.9	36.1 ± 2.4	0.043
	White matter	MW2	30.5 ± 1.7	30.4 ± 3.0	0.927
Gray-White matter Ratio (GWR)					
Basal ganglia			1.30 ± 0.09	1.23 ± 0.07	0.005
Cerebrum			1.23 ± 0.06	1.19 ± 0.07	0.041
Average			1.26 ± 0.07	1.21 ± 0.06	0.003

*CN* caudate nucleus, *PU* putamen, *CC* corpus callosum, *PIC* posterior internal capsule, *MC1* medial cortex at the centrum semiovale, *MW1* medial white matter at the centrum semiovale, *MC2* medial cortex at the high convexity level, *MW2* medial white matter at the high convexity level, GWR Basal ganglia = (CN + PU)/(CC + PIC); GWR Cerebrum = (MC1 + MC2) / (MW1 + MW2); GWR Average = (GWR Basal ganglia + GWR Cerebrum)/2.

### Ratio of gray matter to white matter

**Figure 4:**
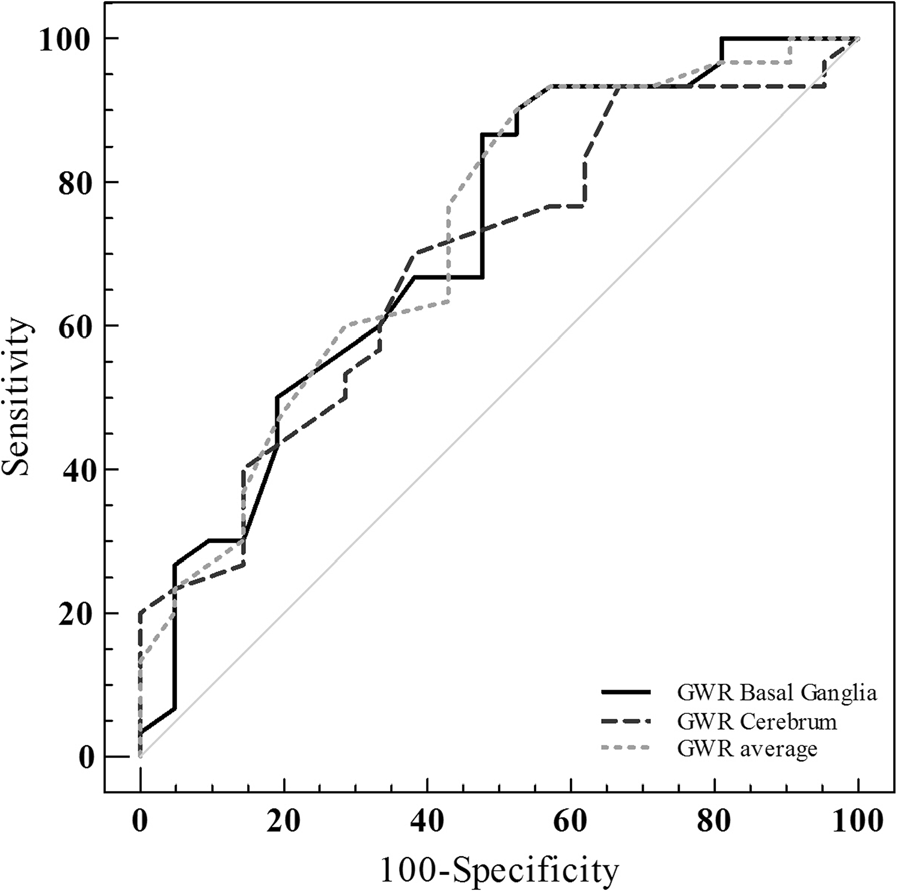
Receiver operator characteristic curve for the prediction poor outcome using the GWRs.

The three GWRs were significantly higher in patients with good neurological outcomes than in patients with poor neurological outcomes (p = 0.005, 0.041 and 0.003, respectively). The average GWR was the strongest predictor of poor neurological outcome as assessed using ROC curves (basal ganglia AUC = 0.716; cerebrum AUC = 0.685; average AUC = 0.747) (Figure 4). Four patients who had an average GWR < 1.14 predicted a poor neurological outcome with a sensitivity of 13.3% (95% confidence interval (CI) 3.8-30.7), a specificity of 100% (95% CI 83.9-100), a positive predictive value of 100% (95% CI 2.5-100), and a negative predictive value of 44.7% (95% CI 28.9-58.9) (Table 3).

**Table 3:** Specificity and sensitivity for poor outcome of attenuation measurements and GWR

**GWR**	**Cutoff value**	**Sensitivity**	**Specificity**	**PPV**	**NPV**	**AUC (95% CI)**	**p**
Basal ganglia	1.12	3.3	100	100	42.0	0.716	0.005
	1.24	66.7	61.9	71.4	56.5	(0.572-0.833)	
Cerebrum	1.12	20.0	100	100	46.7	0.685	0.015
	1.22	70.0	61.9	72.4	59.1	(0.540-0.808)	
Average	1.14	13.3	100	100	44.7	0.747	0.003
	1.24	76.7	57.1	71.9	63.2	(0.579-0.838)	

*GWR *gray-white matter ratio, *PPV *positive predictive value, *NPV *negative predictive value, *AUC *area under curve, *CI* confidence interval.

## Discussion

Post-cardiac arrest brain injury remains a common cause of morbidity and mortality despite the advances made in treatment over time [11]. The measurement of the severity of brain injuries after cardiac arrest is important for the prediction of neurological outcomes. The prediction of the likelihood of neurological recovery for patients in comas is important from several perspectives, such as cost containment, the facilitation of organ donation, and providing information to the patients’ families so that therapeutic decisions can be made.

Brain CT is a useful method to evaluate comatose patients after cardiac arrest because the images are readily obtained within a short period of time. Cocchi M. N. et al. [12] reported that it may be important and useful to obtain a brain CT scan early in the clinical course because the results of such imaging could aid in diagnosis and the selection of the appropriate therapeutic interventions. However the utility of CT scans taken shortly after cardiac arrest not known because few studies have investigated the relationship between the interval and the incidence of CT signs in OHCA survivors. The timing of brain CT scans has not been uniform in previous studies; the intervals range from several hours to days after cardiac arrest [8,9,13,14]. Therefore, the timeline of the initial appearance of recognizable ischemic signs on CT scans is not known. Inamasu et al. [15] reported significant differences in the incidence of loss of boundary signs on CT scans and in the unfavorable outcome rates between scans with a cardiac arrest to ROSC interval ≤20 min and scans with an interval >20 min. The median time from ROSC to the CT scan was only 16 minutes (range, 1 to 59 minutes) in our study, which is very short relative to the median times in previous studies. However, if this time frame is sufficient to identify ischemic brain injury after cardiac arrest, immediate brain CT scans after ROSC will be more helpful for the early determination of the prognosis.

The CT signs of brain ischemia/hypoxia resulting from cardiac arrest are well known [16]. The loss of differential between the GM and WM and cortical sulcal effacement are the signs most frequently documented in the literature [8,9,13,14]. The difference between GM and WM in CT images arises because the higher water content and lower lipid content of the GM result in a higher oxygen concentration and a lower carbon concentration, which increase the level of photoelectric absorption [17]. The selective vulnerability of GM to ischemia results from its higher metabolic rate, greater blood flow, and susceptibility to excitotoxicity [18,19]. Increased vascular permeability leads to the extravasation of fluid (vasogenic edema) and to changes in ion flow due to excitotoxicity (cytotoxic edema), and these factors contribute to the preferential development of edema in the GM over the WM. Significant differences between the two groups in our study were observed in all of the studied GM areas except the MC1.

The CN and PU at the basal ganglia level are commonly used for the measurement of GM attenuation, but the predictive power of GM attenuation for patient prognosis after cardiac arrest is lower than that of the GWR [8]. The same result was observed in our study. Changes in the GWR reflect edema and decreased x-ray attenuation in the GM. Therefore, the GWR is the preferred metric for the identification of loss of boundary signs and the extent of brain injury. The GWR could be used to predict outcomes instead of the attenuation of the GM alone. A recent study revealed that an average GWR < 1.20 exhibited a specificity of 98% for the prediction of mortality, similar to the specificities of previous study cutoffs of 1.18 and 1.22, which were 100% [8-10].

The cutoff value for the GWR was lower in our study when using ROC analysis than in previous studies and a GWR < 1.14 exhibited a specificity of 100% for the prediction of a poor neurologic outcome. The reasons for this difference are not clear. However, all of the patients in our study group received therapeutic hypothermia; therefore, patients in whom a poor neurological prognosis was predicted according to the results of previous studies exhibited a decreased probability of a poor outcome because of the use of therapeutic hypothermia. However, the cutoff value of <1.14 for the GWR exhibited a sensitivity of only 13.3%, which may decrease the power to predict outcomes.

The level of the basal ganglia is the best level for the assessment of GWR [8,9], consistent with the known sensitivity of the basal ganglia to injurious stimuli due to their high metabolic activity and their location within the boundary zones of perfusion. However, Wu et al. [20] reported that edema throughout the brain increases the likelihood of mortality relative to edema in a single region. We measured the GWR at the levels of the basal ganglia and the cerebrum. The GWR varied dependently of each other. The cerebrum GWR was lower on average than the basal ganglia GWR, suggesting that the cerebrum was more prone to edema than the basal ganglia. We also calculated the average of the two GWRs, and this average had the strongest predictive power. These results suggest that the determination of the GWRs of various regions in the whole brain may aid in the prediction of neurological outcomes after cardiac arrest.

This study suffers from several limitations. First, this study was retrospective. Second, the sample of 51 survivors included in this study may not be sufficiently large to achieve high statistical power. Third, patients who did not undergo CT scans were excluded despite the use of therapeutic hypothermia treatment, and this exclusion may have resulted in selection bias and thus influenced the results. Fourth, our cutoff value was statistically significant to determine prognosis, but the sensitivity was low. The prognostic power was not sufficient to predict outcomes. The prognosis was also difficult to predict using the CT results alone. Finally, we did not consider differences in the time between collapse and the CT scans or the different etiologies of arrest, such as cardiac, respiratory and others, in our patients. Some investigators have reported that the time between arrest and the CT scan is not related to the GWR or the attenuation values for any brain region, but the impact of variations in this duration cannot be disregarded. Therefore, a well-designed prospective study using a larger number of patients is required to confirm the GWR cutoff value for the prediction of poor neurological outcomes.

## Conclusion

Our study demonstrated that a low GWR, as determined from immediate brain CT scans in patients treated with therapeutic hypothermia after ROSC, was associated with a poor neurological outcome. And the GWR is very attractive for early outcome prediction because many hospitals treat OHCA patients but not all of them have a specialized neurology service for electrophysiological testing and interpretation of clinical findings. It may be helpful to develop an early prognostic model for cardiac arrest patients treated with therapeutic hypothermia.

## Abbreviations

OHCA: Out-of-hospital cardiac arrest; CT: Computed tomography; GM: Gray matter; WM: White matter; GWR: Gray matter to white matter ratio; ROSC: Return of spontaneous circulation; GCS: Glasgow Coma Scale; CPC: Cerebral Performance Category; CPR: Cardiopulmonary resuscitation; ROI: Regions of interest; HU: Hounsfield Units; CN: Caudate nucleus; PU: Putamen; CC: Corpus callosum; PIC: Posterior limb of the internal capsule; MC: Medial cortex; MW: Medial white matter; ROC: Receiver operating characteristic; AUC: Area under the curve.

## Competing interests

The authors have no competing interests.

## Authors’ contributions

SHK performed data analysis and drafted the manuscript. SPC acquired data and critical revisions to the manuscript. SHO, CSY and KNP managed the data and critical revisions to the manuscript. SMC conceived the research and drafted the manuscript. All authors read and approved the final manuscript.
